# Prevention of Sleep Apnoea After General Anaesthesia Using a Thermoplastic Mandibular Advancement Device: A Randomised, Controlled, Single-Blind Trial

**DOI:** 10.3390/jcm15156042

**Published:** 2026-08-03

**Authors:** Eric Albrecht, Julien Wegrzyn, Jean-Benoit Rossel, Virginie Bayon, Sibylle Chatelain, Yves Renard, Erin Gonvers, Adrien Waeber, Karma Lambercy, Raphaël Heinzer

**Affiliations:** 1 Department of Anaesthesia, University Hospital of Lausanne, University of Lausanne, 1011 Lausanne, Switzerland; yves.renard@chuv.ch (Y.R.); erin.gonvers@chuv.ch (E.G.); 2 Department of Orthopaedic Surgery, University Hospital of Lausanne, University of Lausanne, 1011 Lausanne, Switzerland; julien.wegrzyn@chuv.ch; 3 Centre for Primary Care and Public Health (Unisanté), University Hospital of Lausanne, University of Lausanne, 1011 Lausanne, Switzerland; jean-benoit.rossel@unil.ch; 4 Centre for Investigation and Research in Sleep, University Hospital of Lausanne, University of Lausanne, 1011 Lausanne, Switzerland; virginie.bayon@chuv.ch (V.B.); adrien.waeber@chuv.ch (A.W.); raphael.heinzer@chuv.ch (R.H.); 5 Department of Dentistry, University Hospital of Lausanne, 1011 Lausanne, Switzerland; sybille.chatelain@chuv.ch; 6 Department of ENT and Head and Neck Surgery, University Hospital of Lausanne, 1011 Lausanne, Switzerland; karma.lambercy@chuv.ch

**Keywords:** sleep apnoea, general anaesthesia, orthopaedic surgery, perioperative medicine

## Abstract

**Background:** Sleep apnoea is associated with increased postoperative morbidity and worsens after surgery under general anaesthesia. A thermoplastic mandibular advancement device (MAD) might prevent postoperative sleep apnoea worsening. This randomised, controlled, single-blind trial tested the hypothesis that a thermoplastic MAD would reduce apnoea–hypopnoea index (AHI) on the first postoperative night after general anaesthesia. **Methods:** One hundred patients undergoing various elective orthopaedic procedures on the lower limb under general anaesthesia were randomly allocated to two groups: use of a thermoplastic MAD during the first postoperative night (intervention) or no MAD during the first postoperative night (control). All patients underwent respiratory polygraphy on the first postoperative night. The primary outcome was the AHI on the first postoperative night. Secondary outcomes included the obstructive apnoea index, mixed apnoea index, central apnoea index, hypopnoea index, oxygen desaturation index, and self-reported tolerance of the device (on a numeric rating scale from 0 to 10, where higher scores indicate better tolerability), all measured during the first postoperative night. **Results:** The mean [95% confidence interval] AHI was similar in the MAD and control groups (9 [6–12] and 11 [7–15] events·h^−1^, respectively; *p* = 0.26). There were no significant between-group differences in the secondary outcomes. The median (interquartile range) score for MAD tolerance was 7.5 (5.0, 8.5). **Conclusions:** In conclusion, a thermoplastic MAD did not demonstrate to reduce AHI after general anaesthesia for lower limb orthopaedic surgery on the first postoperative night. However, this finding should be interpreted cautiously, as dexamethasone administered to all participants may have reduced postoperative respiratory events or increased nocturnal wakefulness, thereby artificially lowering the apnoea–hypopnoea index calculated.

## 1. Introduction

Patients with obstructive sleep apnoea (OSA) have increased morbidity after surgery [1,2]. A recent retrospective analysis of more than 349,000 people undergoing hip arthroplasty reported that sleep apnoea syndrome was associated with higher rates of cardiovascular and pulmonary complications, longer hospital stay, and re-admission [2]. The number of apnoea and hypopnoea episodes has been shown to increase significantly up to the third night after surgery whether the procedure is performed under spinal or general anaesthesia [3]. Furthermore, the use of short-acting anaesthetic agents, such as desflurane and remifentanil, does not reduce the number of postoperative sleep apnoeic episodes compared with standard anaesthetic agents, including sevoflurane and fentanyl [4].

A mandibular advancement device (MAD) is a small device that is inserted in the mouth of the patient and protrudes the mandible to prevent upper airway collapse in people with OSA who are unable to tolerate continuous positive airway pressure (CPAP) therapy, thus reducing the number of sleep apnoea episodes. A MAD is usually well tolerated and has been shown to be as effective at reducing the number of apnoeas and hypopnoeas as CPAP in patients with mild to moderate sleep apnoea [5,6]. Thermoplastic MADs can be moulded on the patient’s maxilla and mandible at the bedside and effectively reduce the number of apnoea and hypopnoeas [7]. As suggested in a previous publication, temporary MAD prescription could represent a satisfactory and cost-effective alternative for the postoperative management of people with OSA [4].

Therefore, this study tested the hypothesis that a thermoplastic MAD would reduce apnoea–hypopnoea index (AHI) on the first postoperative night in patients undergoing various elective orthopaedic procedures on the lower limb under general anaesthesia compared with a control group.

## 2. Materials and Methods

This randomised, single-blind, parallel-group trial (NCT04731168) was conducted at the University Hospital of Lausanne between February 2021 and September 2023. Randomisation was undertaken using a computer-generated randomisation table in aggregates of 10. Assignments were concealed in a sealed opaque envelope. The sleep physician and the statistician were not aware of treatment allocation. The study was approved by the hospital ethics committee and conducted according to Good Clinical Practice and Declaration of Helsinki 2002 principles. All patients provided written informed consent, and the study was monitored by an independent person.

Patients were eligible for participation in the study if they were aged over 18 years and scheduled to undergo lower limb elective orthopaedic surgery under general anaesthesia and had a Neck, Obesity, Snoring, Age, Sex (NoSAS) score of ≥8 [8]. Points are awarded for each item and a score of ≥8 indicates a high probability of sleep-disordered breathing. More details are provided in Appendix A. Exclusion criteria included unhealthy teeth condition, mandibular prognathism, temporomandibular joint dysfunction, treatment of sleep apnoea with CPAP, presence of severe respiratory or cardiovascular disease, malignant hyperthermia, preoperative consumption of benzodiazepines, chronic use of opioids at a dosage of ≥30 mg·day^−1^ morphine equivalent, and pregnancy. On the day of surgery, patients were randomised to wear a commercial thermoplastic MAD (Somnofit, Oscimed SA, La Chaux-de-Fonds, Switzerland) during the first postoperative night (MAD group) or not (control group).

Anaesthesia was induced using intravenous propofol 1.5–2 mg·kg^−1^ and sufentanil 0.1 μg·kg^−1^, with endotracheal intubation facilitated by intravenous rocuronium 0.6 mg·kg^−1^. Anaesthesia was maintained using sevoflurane in an air–oxygen mixture at a concentration of 0.8–1.2 MAC (minimum alveolar concentration) to achieve a bispectral index between 40 and 60 (monitoring of anaesthesia depth). Analgesia to manage increases in heart rate or blood pressure of more than 20% above preoperative values was provided with bolus 2.5 µg doses of sufentanil. Positive pressure ventilation was initiated, and tidal volume and rate were adjusted to maintain end-tidal carbon dioxide pressure at 35 to 40 mmHg. As per routine institutional practice, all patients received intravenous dexamethasone 0.1–0.2 mg·kg^−1^ at the beginning of surgery, and acetaminophen 1000 mg, ketorolac 30 mg and ondansetron 4 mg at the end of surgery for multimodal analgesia and antiemetic prophylaxis. In case of residual neuromuscular blockade (defined as a train-of-four ratio <0.9), muscle relaxation was antagonised with neostigmine 50 μg·kg^−1^ and glycopyrrolate 5–10 μg·kg^−1^ [9]. In phase I recovery, pain was assessed on a visual analogue scale from 0 to 10. A score of 4 or more, or patient request for analgesia, was managed with morphine 2 mg every 10 min as needed. After resumption of oral intake, patients received acetaminophen 1000 mg every 6 h, ibuprofen 400 mg every 6 h, oral controlled-release morphine 20 mg every 12 h and immediate-release morphine 10 mg every 4 h as needed. Ongoing antiemetic medication included intravenous ondansetron 4 mg as needed. Patients received oxygen at a rate of 2–4 L·min^−1^ in phase 1 recovery but not after transfer to the ward.

Sleep-related parameters and outcomes were measured on the first postoperative night after surgery using a portable respiratory polygraphy recorder (ApneaLink Air^TM^, San Diego, CA, USA). This system, previously validated against polysomnography allows a non-invasive recording of nasal airflow via nasal cannula, oxygen saturation using finger pulse oximetry, and respiratory efforts using a thoracic belt [10]. All recordings were scored by a sleep specialist who was unaware of treatment allocation. Apnoea was defined as breathing cessation for ≥10 s, and hypopnoea was defined as a fall of ≥30% in the respiratory flow signal associated with a ≥3% drop in oxygen saturation. The AHI was defined as the number of apnoeas and hypopnoeas per hour of recording time, and the oxygen desaturation index (ODI) reflected the number of oxygen desaturations of ≥3% per hour of recording time. Central sleep apnoea was defined as the presence of >50% of events without thoracic movements. Before setting up the portable respiratory polygraphy recorder, a thermoplastic MAD (Somnofit^®^-S, Oscimed SA) was placed in a warm water bath for 2 min then inserted in the patient’s mouth. The research assistant, who had been trained by a dentist, moulded the impression of the patient’s maxilla and mandible, then adjusted the device.

The primary outcome was the AHI on the first postoperative night. Secondary sleep-related outcomes were the obstructive apnoea index, mixed apnoea index, central apnoea index, hypopnoea index, ODI, mean oxygen saturation, percentage of recording time with oxygen saturation below 90%, and hypoxic burden, all on the first postoperative night. Other secondary outcomes were tolerance of the MAD (rated on a numeric analogue scale from 0 to 10, where higher scores indicate better tolerability), sleep quality (rated on a numeric analogue scale from 0 to 10, where higher scores indicate better sleep quality), and Stanford sleepiness scale (a 7-point Likert scale where higher scores indicate greater sleepiness).

It was calculated that 41 patients were required in each treatment group to detect a difference of 10 events·h^−1^ in the AHI with a standard deviation of 16, 80% power and a two-sided alpha error of 0.05. Based on an estimated drop-out rate of 20%, the recruitment target was set at 100 subjects (50 per group).

A modified (in case of missing primary outcomes) intention-to-treat statistical analysis was performed using Stata 18 software (StataCorp 2023, Stata Statistical Software: Release 18, College Station, TX, USA). Additional sensitivity analyses were also performed, including as-treated (according to the treatment patients received rather than the one to which they were randomised) and per protocol (restricted to patients who adhered to the protocol as specified) analyses. Categorical data are summarised as rates, while continuous data are reported as mean with 95% confidence interval (CI) values or median and interquartile range (IQR). Categorical data are compared using Fisher’s exact test or Pearson’s Chi-square test, as appropriate. Normality of continuous data was checked with the Shapiro–Wilk test. Continuous variables were analysed using an independent *t*-test or Mann–Whitney U test, as appropriate. Significance was considered at *p* < 0.05 based on a two-tailed probability.

## 3. Results

**Figure 1:**
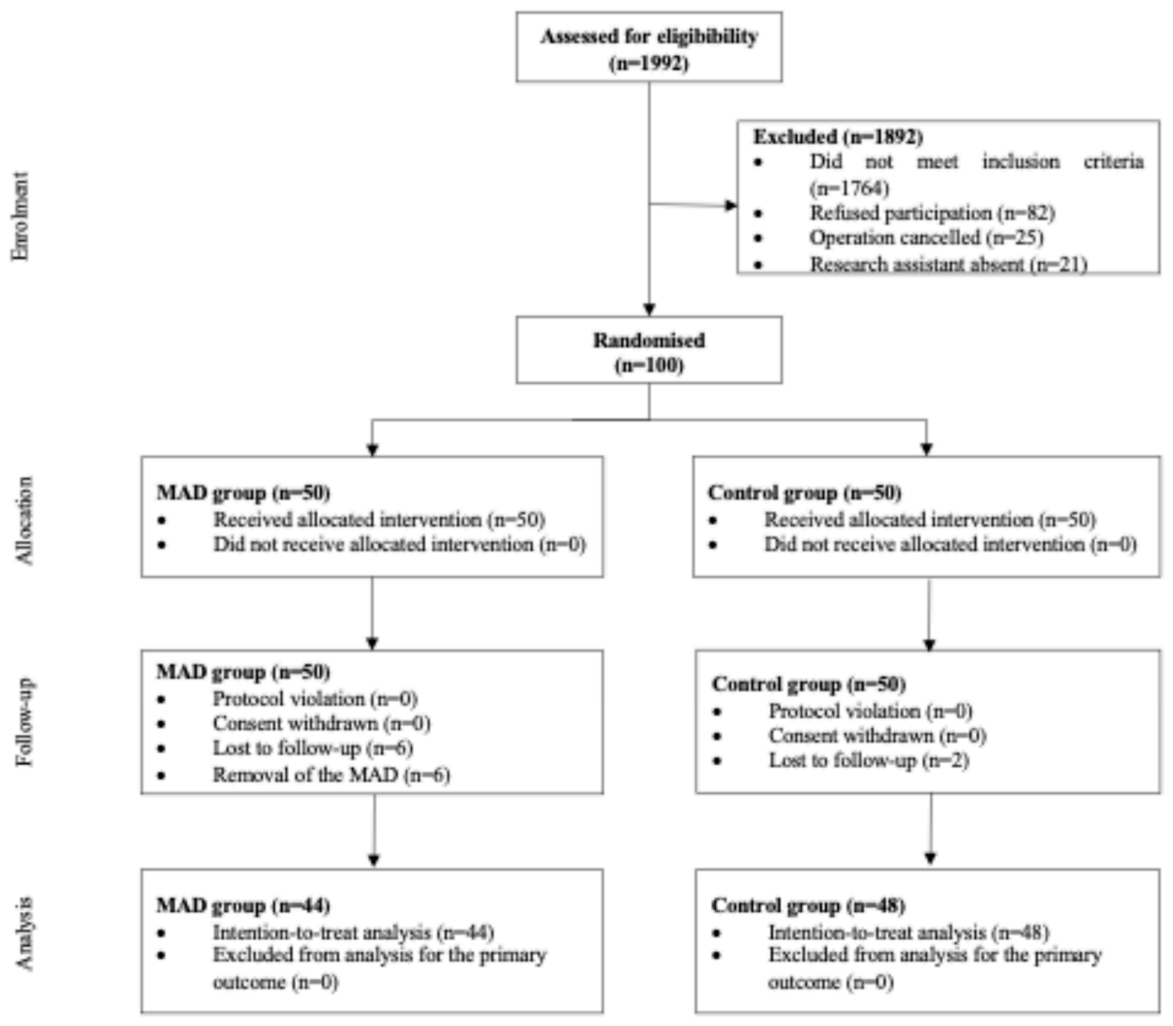
A flowchart of the study population.

Among the 1992 patients assessed for eligibility, 100 individuals were recruited; an important majority (*n* = 1764) did not meet the inclusion criteria, the most frequent reason being a NoSAS score < 8. Eight participants had technical issues with the portable respiratory polygraphy device and therefore 92 patients (44 in the MAD group and 48 in the control group) had available data for analysis of the primary outcome (Figure 1). Six of the 50 patients in the MAD group with available data removed the device after 1–2 h due to jaw discomfort. The median (IQR) score for device tolerance was 7.5 (5.0, 8.5), indicating good tolerability.

**Figure 2:**
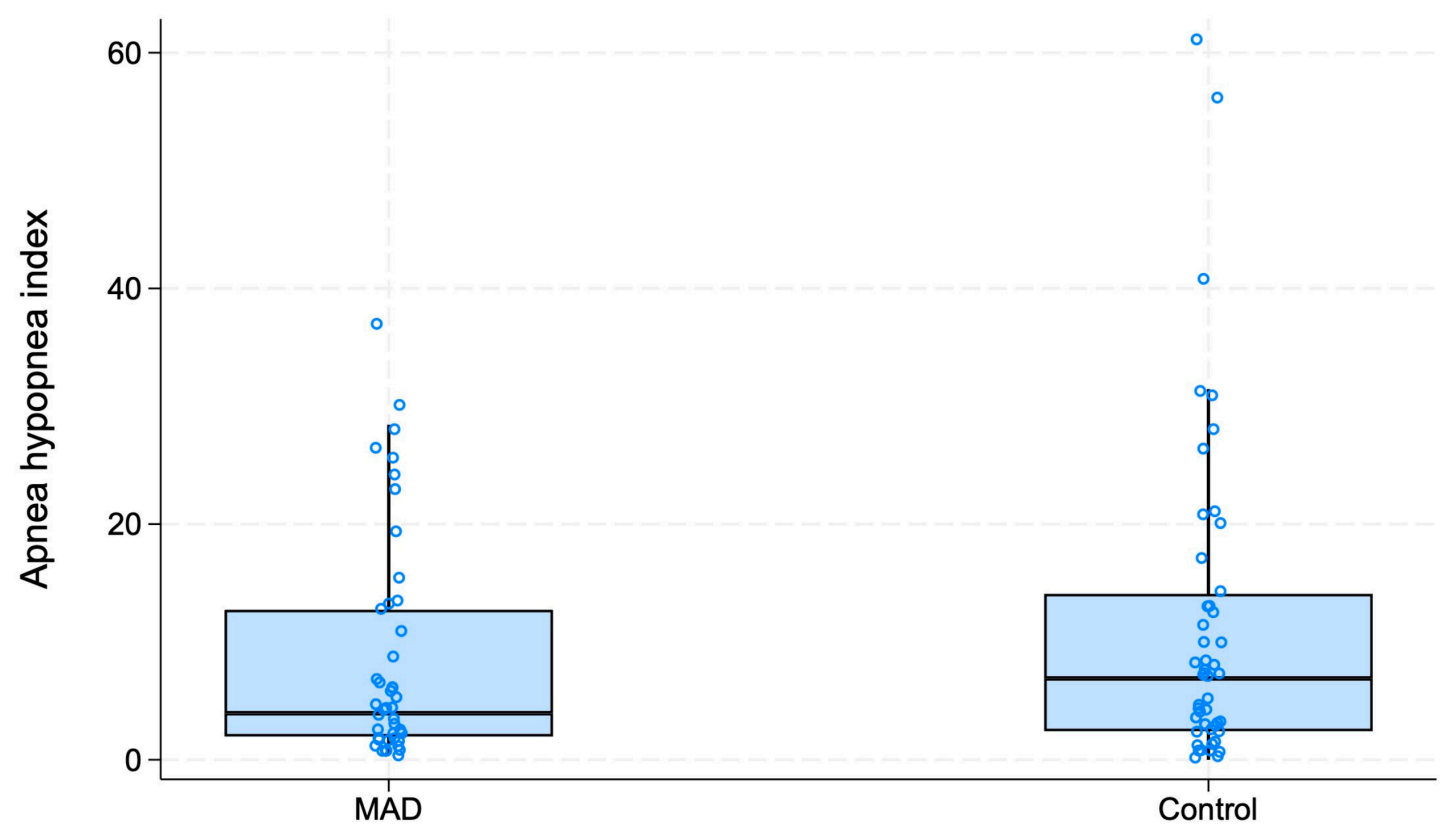
A box plot of the primary outcome.

The two study groups were well matched at baseline (Table 1). The mean [95% CI] AHI on the first postoperative night (primary outcome) was similar in the MAD and control groups (8.6 [5.6–11.5] and 11.4 [7.4–15.4] events·h^−1^; mean difference: −2.8 [−7.8–2.2]; *p* = 0.262) (Figure 2). There were also no significant differences between groups in the other sleep study data (Table 2). Table A2 and Table A3 present the results after as-treated and per protocol analyses.

## 4. Discussion

This randomised controlled trial investigating whether a MAD would reduce the number of apnoea and hypopnoea episodes in at-risk individuals with OSA on the first postoperative night after lower limb elective orthopaedic surgery under general anaesthesia failed to reject the null hypothesis. The mean AHI during the first postoperative night was similar in both the MAD and control groups (9 and 11 events·h^−1^, respectively). These data did not demonstrate that the convenient and economical use of a MAD is effective in reducing the AHI in a surgical setting. However, there was an interesting trend towards a reduction in the time spent with oxygen desaturation of below 90% in the MAD versus control group (22% vs. 36%, *p* = 0.06), and we cannot exclude the possibility that this between-group difference might have achieved statistical significance with a larger sample size. Oxygen desaturation/hypoxia is potentially clinically relevant because this parameter has been reported to be associated with the risk of stroke and major adverse cardiovascular events in several cohorts [11,12]. More specifically, one of these two studies concluded that in patients with newly diagnosed obstructive sleep apnoea and no established cardiovascular disease, greater OSA-specific hypoxic burden and nocturnal hypoxaemia were independently associated with a higher risk of major cardiovascular events and mortality [12].

Our short-term negative results differ from those of a previous trial that evaluated the longer-term use of a MAD at home in 315 participants [7]. In that trial, the 2-year success rate of therapy with a custom-made MAD (defined as a ≥50% reduction in the AHI from baseline) was 67%. There are several possible explanations for the different findings between the study, including the different MADs used (thermoplastic versus custom-made), the short- versus long-term nature of the study, and the different populations (general sleep apnoea population in the previous trial and post-surgery individuals at risk of OSA in the current study). In addition, 12% of the participants in our study removed the MAD during the first postoperative night. An option to improve device usage on the first night would be to provide surgical patients at risk of OSA with a custom-made MAD from a dentist, which should be more comfortable, and to instruct them to use the device at home a few days before surgery so that they could get accustomed to it. In addition to potentially improving tolerability, this could also achieve more effective reduction in the AHI.

Another explanation for the neutral primary outcome finding of our study might be the low AHI in the control group (11 events·h^−1^). This is substantially lower than in one of our previous studies where the mean AHI during the first postoperative night was 20 events·h^−1^ (95% CI 15–24) [3]. On that basis, the current study was powered to detect a between-group difference of approximatively 10 events·h^−1^. The lower AHI in the control group could have been due to several factors. First, we included patients scheduled for all types of elective lower limb orthopaedic surgery as opposed to elective hip arthroplasty, and therefore the sample population in the trial is 10 years younger than in our previous trial (age 55 vs. 65 years). Second, we used a different portable respiratory polygraphy recorder (ApneaLink Air^TM^ vs. Embletta^®^), but this should not have had an important impact on our findings because both devices were validated by the same group of authors using the same methodology, even if this device did not record the body position [10,13]. Another limitation is the use of respiratory polygraphy rather than full polysomnography, which prevented assessment of sleep architecture and differentiation between sleep and wakefulness during the recording period. Dexamethasone was given at the beginning of surgery in all participants in this trial but not the previous one, and this may have reduced the number of apnoea and hypopnoeas due its possible impact on the upper airway post-intubation, oedema and inflammation [14]. This hypothesis needs to be confirmed in a subsequent trial because dexamethasone could be a cost-effective pharmacological agent to prevent sleep apnoea worsening in the postoperative period, which is also partly due to altered ventilatory control and peripheral chemoreflex activation [15]. Another possibility is that dexamethasone might have contributed to patients having more awake time during the night due to its stimulant effect, which may have artificially reduced the AHI (due to their being now apnoeas and hypopneas during awake time). Unfortunately sleep and wake time were unable to be differentiated in this study and the AHI was calculated based on total recording time.

The main limitation of this study is the absence of recording of the body position with the portable respiratory polygraphy recorder that we used. The proportion of time spent in the supine position (which tends to worsen OSA severity) may have varied between participants and it was not possible to control for this parameter due to a change of the recording device. As a result, we could not measure the predefined primary outcome, supine AHI, and used global AHI instead. However, because all participants underwent lower limb surgery, they probably spent more than 95% of sleep time in the supine position, as previously reported [4]. Therefore, we believe this limitation is unlikely to have materially affected our results. Furthermore, we had some technical issues with the portable respiratory polygraphy recorder 8% of the enrolled participants. However, the number of individuals with analysable sleep exam data remained higher than that calculated in our power analysis, reducing the likelihood of a type II error. That said, we acknowledge that the study might probably be still underpowered for the primary endpoint because the observed between-group difference in AHI was substantially smaller than the difference assumed in the power calculation. Another limitation is incomplete adherence to the mandibular advancement device. Although overall tolerance was rated as good, six of the 50 patients (12%) in the intervention group removed the device after only one to two hours because of jaw discomfort. Consequently, these patients were untreated for most of the night, which may have reduced the effective exposure to the intervention and diluted its potential impact on the primary and secondary outcomes. This rate of device removal may be due to the “thermoplastic” type of device that was used in this study. We cannot exclude that a better fitted “custom made” device could have yielded better results. Finally, due to differences in breathing control physiology between males and females, we cannot exclude sex-related differences in OSA treatment response. However, given the limited proportion of women included, the study was not designed or powered to evaluate such sex-specific effects.

In conclusion, a thermoplastic MAD did not demonstrate to reduce the AHI on the first postoperative night after general anaesthesia in patients undergoing elective lower limb orthopaedic surgery. However, this finding should be interpreted cautiously, as dexamethasone administered to all participants may have reduced postoperative respiratory events or increased nocturnal wakefulness, thereby artificially lowering the apnoea–hypopnoea index calculated from total recording time. These findings therefore require confirmation in a larger trial without routine dexamethasone administration and with objective differentiation between sleep and wake periods. Furthermore, the use of dexamethasone as a cost-effective pharmacological measure to reduce worsening of sleep apnoea in a perioperative setting remains a hypothesis that warrants further investigation. Another limitation is that respiratory polygraphy rather than full polysomnography was used, precluding assessment of sleep architecture and differentiation between sleep and wakefulness; consequently, calculating the apnoea–hypopnoea index using total recording time may have underestimated its true value.

## Figures and Tables

**Table 1:** Baseline demographic and clinical characteristics of study participants.

Characteristics	MAD Group (*n* = 44)	Control Group (*n* = 48)
Age, years	57 (40, 63)	58 (47, 66)
Male sex, *n* (%)	34 (77.3%)	38 (79.2%)
Weight, kg	90 (79, 101)	87 (78, 94)
Height, cm	174 (170, 183)	173 (170, 178)
Body mass index, kg·m^−2^	28 (26, 33)	29 (26, 31)
Neck circumference (cm)	41 (40, 44)	42 (40, 45)
NoSAS score	11 (9, 14)	13 (11, 15)
ASA class, *n* (%)		
I	4 (9%)	10 (21%)
II	36 (82%)	32 (67%)
III	4 (9%)	6 (12%)
Duration of surgery, min	107 (77, 153)	94 (68, 134)
Type of surgery		
Hip arthroplasty	7 (15.9%)	8 (16.7%)
Knee arthroplasty	10 (22.7%)	12 (25.0%)
Knee arthroscopy	1 (2.3%)	6 (12.5%)
Osteotomy	7 (15.9%)	5 (10.4%)
Foot surgery	9 (20.5%)	8 (16.7%)
Other	10 (22.7%)	9 (18.8%)

Data are presented as median (interquartile range) or number of patients (%). AHI, apnoea–hypopnoea index; ASA, American Society of Anesthesiologists; MAD, mandibular advancement device; NoSAS, Neck, Obesity, Snoring, Age, Sex.

**Table 2:** Sleep study data.

	MAD Group (*n* = 44)	Control Group (*n* = 48)	Mean Difference	*p* Value
Obstructive apnoea index, events·h^−1^	0.2 (0.1 to 0.4)	1.5 (0.2 to 2.8)	−1.2 (−2.6 to 0.1)	0.073
Central apnoea index, events·h^−1^	0.7 (0.3 to 1.1)	0.6 (0.1 to 1.1)	0.2 (−0.5 to 0.8)	0.627
Mixed apnoea index, events·h^−1^	0.0 (−0.0 to 0.1)	0.0 (−0.0 to 0.0)	0.0 (−0.0 to 0.1)	0.127
Hypopnoea index, events·h^−1^	7.6 (4.8 to 10.3)	9.4 (6.3 to 12.4)	−1.8 (−5.9 to 2.3)	0.383
Oxygen desaturation index, events·h^−1^	16.2 (12.1 to 20.3)	19.7 (14.6 to 24.7)	−3.4 (−9.9 to 3.0)	0.294
Mean oxygen saturation, %	91.3 (90.7 to 92.0)	90.5 (89.6 to 91.3)	0.9 (−0.2 to 1.9)	0.093
Time with oxygen saturation <90%, %	21.8 (13.5 to 30.0)	34.2 (24.2 to 44.2)	−12.5 (−25.4 to 0.5)	0.059
Hypoxic burden, %min·h^−1^	58.7 (47.7 to 69.6)	60.8 (46.9 to 74.6)	−2.1 (−19.7 to 15.6)	0.814
Stanford sleepiness scale (numeric rating scale, 0–7)	5.2 (4.5 to 5.9)	5.5 (4.9 to 6.2)	−0.3 (−1.3 to 0.7)	0.521
Sleep quality (numeric rating scale, 0–10)	6.8 (6.1 to 7.5)	7.0 (6.3 to 7.7)	−0.2 (−1.1 to 0.7)	0.666

Data are presented as mean with 95% confidence interval.

## Data Availability

The data are available from the corresponding author upon reasonable request.

## Abbreviations

AHI	apnoea–hypopnoea index
CPAP	continuous positive airway pressure
ODI	oxygen desaturation index
OSA	obstructive sleep apnoea
MAD	mandibular advancement device
MAC	minimum alveolar concentration
NoSAS score	Neck, Obesity, Snoring, Age, Sex

## Appendix A. Online Supporting Information

### Summary of the NoSAS Score (NoSAS, Neck, Obesity, Snoring, Age, Sex)

**Table A1:** NoSAS score (a score of ≥8 indicates a high probability of sleep apnea).

	Points
**N**eck circumference > 40 cm	4
**O**besity	
Body mass index 25 to <30 kg/m^2^	3
Body mass index 30 kg/m^2^	5
**S**noring	2
**A**ge > 55 years	4
**S**ex: male	2

**Table A2:** Sleep Study Data Following an As-Treated Analysis.

	MAD Group (*n* = 38)	Control Group (*n* = 54)	Mean Difference	*p* Value
Apnoea hypopnea index, events·h^−1^	9.2 (5.9 to 12.6)	10.6 (7.0 to 14.2)	−1.4 (−6.5 to 3.7)	0.582
Obstructive apnoea index, events·h^−1^	0.3 (0.1 to 0.4)	1.3 (0.1 to 2.5)	−1.0 (−2.4 to 0.3)	0.137
Central apnoea index, events·h^−1^	0.7 (0.3 to 1.1)	0.6 (0.1 to 1.0)	0.1 (−0.5 to 0.8)	0.663
Mixed apnoea index, events·h^−1^	0.1 (−0.0 to 0.1)	0.0 (−0.0 to 0.0)	0.0 (−0.0 to 0.1)	0.074
Hypopnoea index, events·h^−1^	8.2 (5.1 to 11.3)	8.7 (6.0 to 11.5)	−0.6 (−4.7 to 3.6)	0.789
Oxygen desaturation index, events·h^−1^	16.9 (12.2 to 21.5)	18.8 (14.3 to 23.4)	−2.0 (−8.5 to 4.6)	0.558
Mean oxygen saturation, %	91.2 (90.5 to 91.9)	90.7 (89.9 to 91.4)	0.5 (−0.5 to 1.6)	0.312
Time with oxygen saturation <90%, %	23.6 (14.4 to 32.9)	31.5 (22.3 to 40.7)	−7.9 (−21.2 to 5.4)	0.243
Hypoxic burden, %min·h^−1^	58.9 (46.5 to 71.2)	60.4 (47.9 to 72.9)	−1.5 (−19.4 to 16.4)	0.870
Stanford sleepiness scale (numeric rating scale, 0–7)	5.4 (4.7 to 6.2)	5.4 (4.7 to 6.0)	0.1 (−0.9 to 1.0)	0.919
Sleep quality (numeric rating scale, 0–10)	6.8 (6.1 to 7.6)	6.9 (6.3 to 7.6)	−0.1 (−1.1 to 0.9)	0.832

Data are presented as mean with 95% confidence interval.

**Table A3:** Sleep Study Data Following a Per Protocol Analysis.

	MAD Group (*n* = 38)	Control Group (*n* = 48)	Mean Difference	*p* Value
Apnoea hypopnea index, events·h^−1^	9.2 (5.9 to 12.6)	11.4 (7.4 to 15.4)	−2.2 (−7.5 to 3.1)	0.418
Obstructive apnoea index, events·h^−1^	0.3 (0.1 to 0.4)	1.5 (0.2 to 2.8)	−1.2 (−2.7 to 0.3)	0.104
Central apnoea index, events·h^−1^	0.7 (0.3 to 1.1)	0.6 (0.1 to 1.1)	0.2 (−0.5 to 0.8)	0.641
Mixed apnoea index, events·h^−1^	0.1 (−0.0 to 0.1)	0.0 (−0.0 to 0.0)	0.0 (−0.0 to 0.1)	0.095
Hypopnoea index, events·h^−1^	8.2 (5.1 to 11.3)	9.4 (6.3 to 12.4)	−1.2 (−5.5 to 3.2)	0.588
Oxygen desaturation index, events·h^−1^	16.9 (12.2 to 21.5)	19.7 (14.6 to 24.7)	−2.8 (−9.7 to 4.1)	0.425
Mean oxygen saturation, %	91.2 (90.5 to 91.9)	90.5 (89.6 to 91.3)	0.8 (−0.4 to 1.9)	0.182
Time with oxygen saturation <90%, %	23.6 (14.4 to 32.9)	34.2 (24.2 to 44.2)	−10.6 (−24.3 to 3.2)	0.130
Hypoxic burden, %min·h^−1^	58.9 (46.5 to 71.2)	60.8 (46.9 to 74.6)	−1.9 (−20.7 to 16.9)	0.844
Stanford sleepiness scale (numeric rating scale, 0–7)	5.4 (4.7 to 6.2)	5.5 (4.9 to 6.2)	−0.1 (−1.1 to 0.9)	0.810
Sleep quality (numeric rating scale, 0–10)	6.8 (6.1 to 7.6)	7.0 (6.3 to 7.7)	−0.2 (−1.1 to 0.8)	0.750

Data are presented as mean with 95% confidence interval.
